# Surgical treatment for right-side aortic arch concomitant with Kommerell’s diverticulum: techniques selection and follow-up results

**DOI:** 10.1186/s40001-023-01595-5

**Published:** 2024-01-03

**Authors:** Yali Wang, Shuchun Li, Min Jin, Yunxing Xue, Dongjin Wang, Qing Zhou

**Affiliations:** 1 Department of Thoracic and Cardiovascular Surgery, The Affiliated Drum Tower Hospital of Nanjing University Medical School, Nanjing, Jiangsu Province China; 2 Institute of Cardiothoracic Vascular Disease, Nanjing University, Nanjing, China

**Keywords:** Kommerell’s diverticulum (KD), Right aortic arch, Castor, Type B aortic dissection, Surgery technique

## Abstract

**Background:**

Right-side aortic arch concomitant with Kommerell’s diverticulum (KD) is a rare and complex ailment, and there is no consensus on the optimal strategy to deal with this congenital anomaly. We retrospectively analyzed and summary of the cases treated in our center with individual treatment methods for different situations.

**Methods:**

Between September 2018 and December 2021, 10 patients experienced surgical therapy at our institution who presented with a Kommerell’s diverticulum arising from an aberrant subclavian artery from the right-side aortic arch. Four main surgical techniques were applied to those patients: 1. total arch replacement with frozen elephant trunk implantation (*n* = 2); 2. hybrid procedure combining open arch repair and endovascular intervention (*n* = 1); 3. total endovascular repair using thoracic endovascular aortic repair (TEVAR) with or without left subclavian artery (LSCA) revascularization (*n* = 6); 4. direct repair underwent endoaneurysmorrhaphy. Clinical characteristics and outcomes were collected.

**Results:**

The mean age of these 10 patients was 56.5 years (range 29–79 years) and only 1 woman. The pathology includes aortic dissection (*n* = 6) and aneurysm (*n* = 4). The mean diverticulum size was 41.4 [24.2–56.8] mm. There were no in-hospital deaths, and the median hospital stay was 22 [15–43] days. During the follow-up period (21.4 months, 1–44 months), one died of an unknown cause and one died of esophageal fistula. Two patients underwent second-stage endovascular intervention for distal lesion. And none of the patients had endoleak during the follow-up period.

**Conclusions:**

Each of the procedures we have mentioned here has its advantages and disadvantages; individualized treatment should meet the appropriate indications. A single-branched stent graft is feasible and effective in the treatment of aortic disease combined with Kommerell’s diverticulum.

## Introduction

Right-side aortic arch is an uncommon deformity that only occurs in 0.1% of the population [1]. Aberrant subclavian artery pathologies associated with KD, named after Kommerell, who first described it in a patient in 1936, is rarely exist, only about 0.04–0.4% [2, 3]. Moreover, Right aortic arch, aberrant left subclavian artery (ALSA) concomitant with KD are two congenital arch anomalies that extremely rarely happen together. In spite of their rarity, such complex congenital anomalies are more likely to have a significant risk of aortic dissection or rupture [4]. Based on the high mortality rate of the disease, open surgical repair has been the standard treatment in the past, whereas in recent years, other treatment options have been proposed, such as hybrid or endovascular procedures. Unfortunately, there is no consensus on the preferred strategy to treat congenital anomalies of the aortic arch involving Kommerell aneurysm.

The goal of this study is to fill this knowledge gap by presenting our own experience with different approaches through reporting our follow-up outcome in these patients, and assessing prognosis to guide future treatment for patients who underwent open surgical, hybrid, and endovascular management at our center.

## Methods

### Patients and data collection

This is a single-center, retrospective cohort study. Between September 2018 and December 2021, 10 patients underwent surgical therapy at our institution who presented with a KD arising from an aberrant subclavian artery from the right aortic arch. All patients were preoperatively documented to have an abnormal aortic arch and a KD identified either by radiological or echocardiography. Clinical data of all patients were collected and enrolled from the Department of Cardiothoracic Surgery of Affiliated Drum Tower Hospital of Nanjing University Medical School and follow-up outcome data obtained by reexamine on outpatient clinic visits 3 months after surgery. Our proposals about this study were reviewed and approved by the institutional review board.

### Preoperative computed tomography (CT) assessment and measurement

Preoperative neck and thoracoabdominal aorta enhance CT were performed in all instances to assess the size of Kommerell’s diverticulum and select proper operative approaches. Preoperative and postoperative CT scans were analyzed using centerline reconstruction to obtain accurate KD measurements. The size of KD was measured in the maximum diameter of the originating of the subclavian artery by Endo Size. And follow-up result analysis was also performed on enhanced CT scans.

Right-side arch with ALSCA [5] is defined as the first branch to arise from this right-sided arch is left common carotid artery (LCCA), followed by the right common carotid artery (RCCA) and then the right subclavian arteries (RSA), the fourth branch is left subclavian artery (LSCA) isolation arising from arch.

### Surgical approach

#### Strategy 1: Total arch replacement combining with frozen elephant trunk implantation in a single stage

**Fig. 1:**
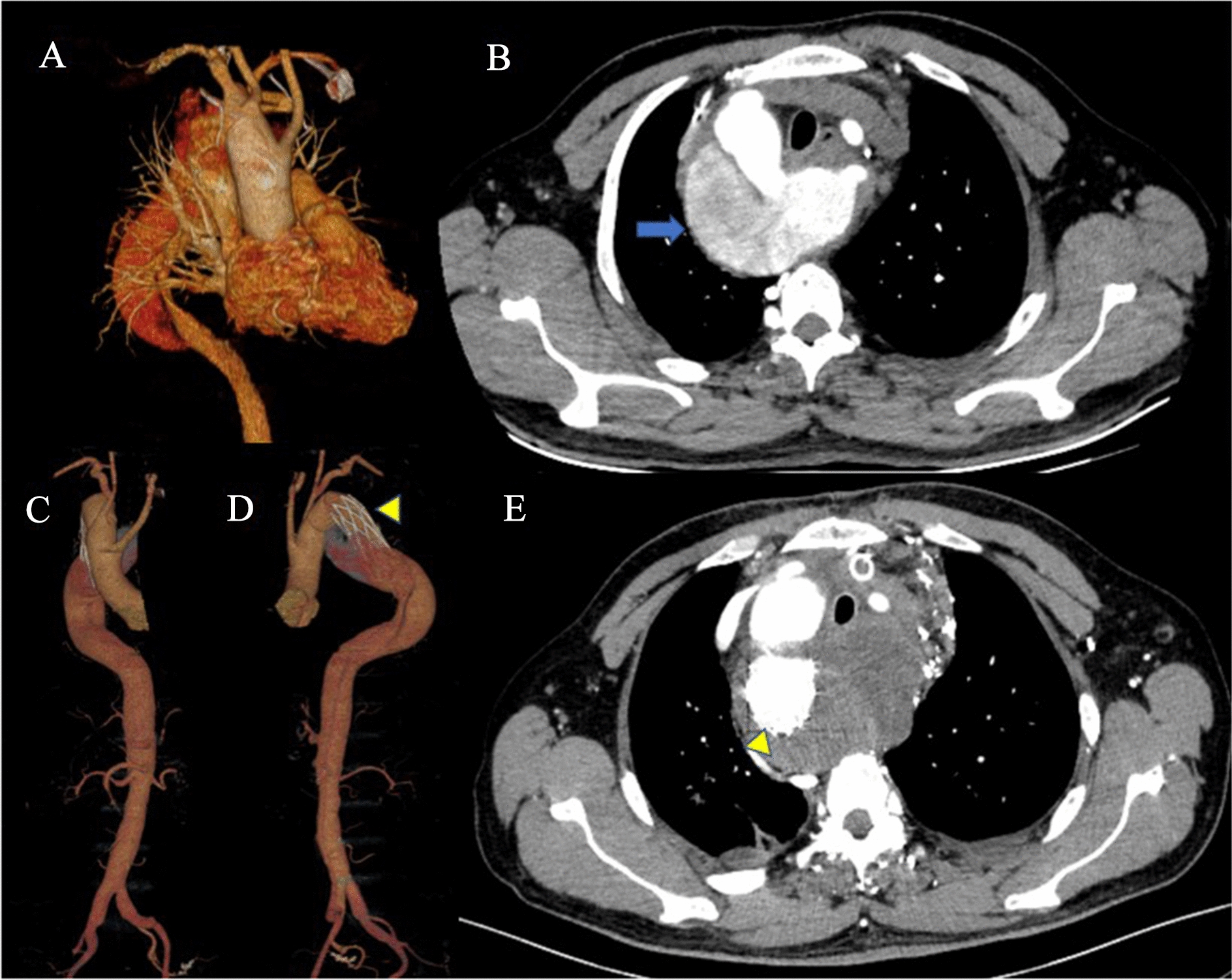
Surgical schema of total arch replacement with frozen elephant trunk implantation via median sternotomy. Three-dimensional reconstruction and CT scan of a right-sided arch with a Stanford B type aortic dissection involving the KD with aberrant LSA (**A** and **B**); the dilation aortic aneurysm (blue arrow) was opened and descending aortic replacement was completed (yellow arrowhead). Three-dimensional reconstruction image of postoperative computed tomographic angiogram shown in different direction. **C**,** D** The abdominal aortic dissection was repaired in the second stage

The supra-arch vessels and ALSCA were exposed through median sternotomy, ALSCA usually runs behind the esophagus and trachea to the left. Cardiopulmonary bypass (CPB) was established through ascending aorta and right atrium cannulation. With the patient under deep hypothermia of 22–24 ℃, the right aortic arch was opened under circulatory arrest. A frozen elephant trunk CRONUS ® graft (MicroPort Shanghai) was installed into descending aorta to cover the ostium of KD. The remained supra-arch vessels were all anastomosed with the four branches Dacron graft (Fig. 1).

#### Strategy 2: Hybrid procedure combining open arch repair (without cardiopulmonary bypass) and endovascular stent implantation

**Fig. 2:**
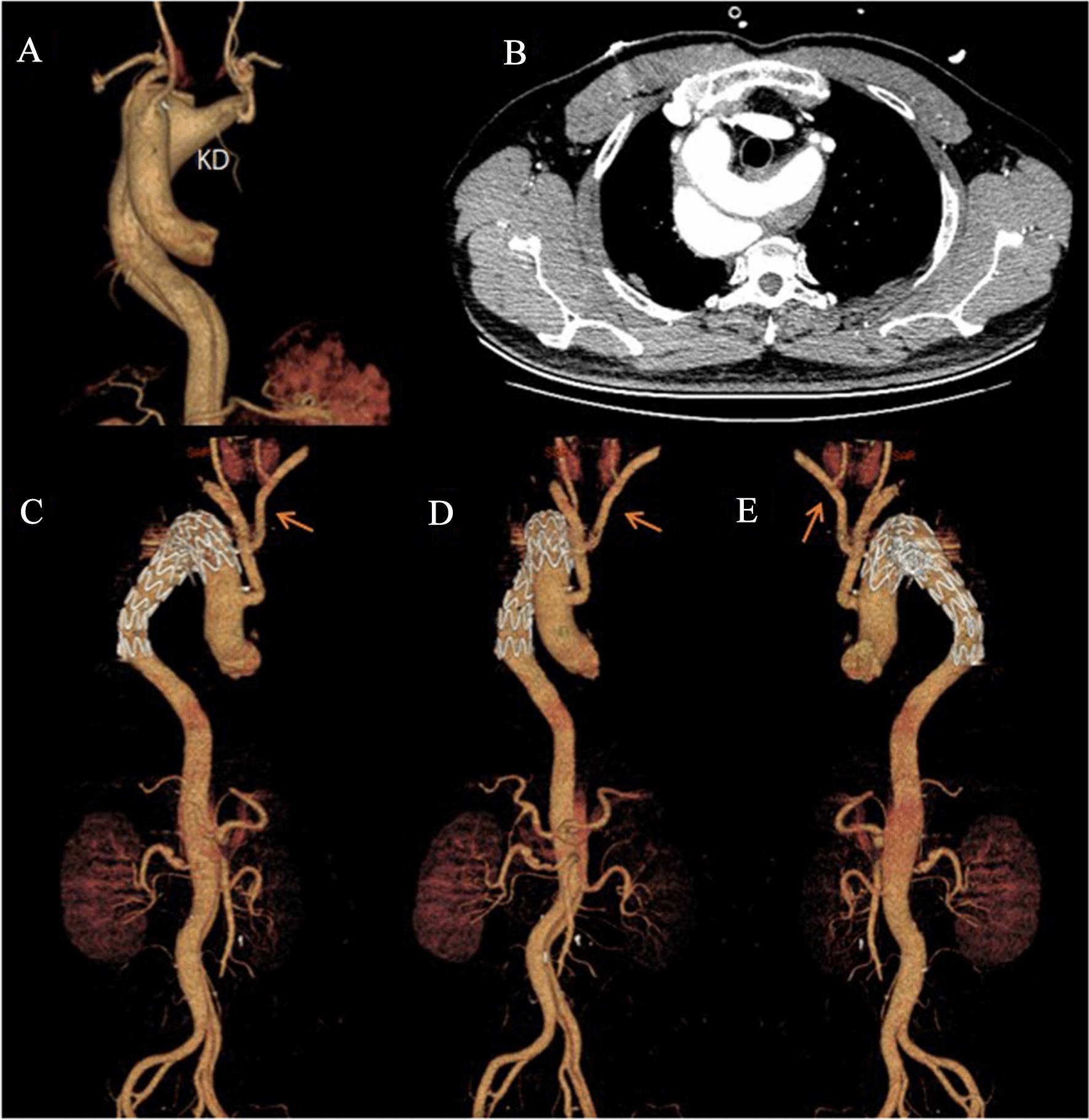
Hybrid procedure combining open arch repair and endovascular intervention. 3D reconstruction and preoperative CT scan of a right-sided arch with a Stanford B type aortic dissection involving the KD with aberrant LSA (**A**, **B**); reconstruction image of postoperative computed tomographic angiogram shown in different direction (**C**–**E**) and anastomosing branched vessels by Y-shape graft (orange arrow)

The supra-arch vessels were exposed through median sternotomy; left and right carotid and right subclavian artery were ligated proximal to the origin of the aortic arch. The proximal anastomosis of debranching graft was constructed after the proximal ascending artery was side-clamped. A Y-graft was performed end-to-side to the aorta arch which was constructed using a 10-mm Dacron graft (Terumo, Japan). Next, the right subclavian artery, LCCA and RCCA were anastomosed end–end to the main body of the debranching graft by a Y-graft. Last, the left subclavian artery was anastomosed end-side to the LCCA using a 10-mm Dacron grafts. TEVAR (Ankura, Lifetech) was performed and deployed to overlap the KD through right femoral artery after the branch repair was complete. If there was an endoleak that developed after the aortogram was finished, the opening of KD was occluded by coil embolization (Cook, USA) (Fig. 2).

#### Strategy 3: Total endovascular repair with or without LSCA revascularization

**Fig. 3:**
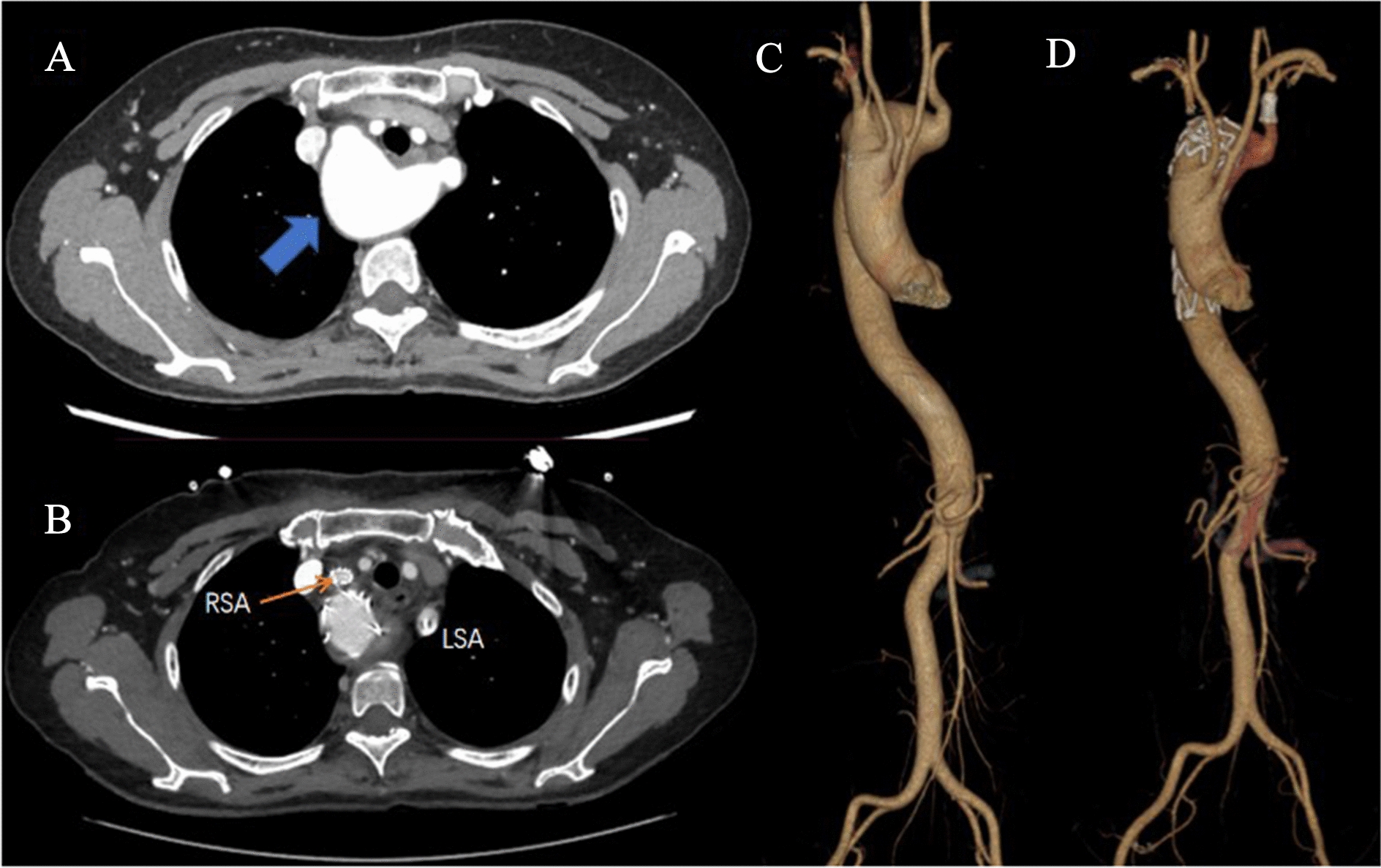
Surgical schema of Castor via femoral approach. **A**, **B** The changes of lesion on CT before and after operation. A right-side arch concomitant with KD (blue arrow) was repaired by Castor, in which the branch stent was placed in the RSA (orange arrow). And we can clearly see the LSA where the coil was embolized (**B**). Three-dimensional reconstruction image of preoperative (**C**) and postoperative (**D**) computed tomographic angiogram was shown

The LSCA and LCCA were exposed through a supra-clavicular incision; an 8-mm Dacron graft was used to anastomosed to the LSCA and LCCA in an end-to-side manner except for one patient (patient 3) with left vertebral dominance. After aortography, the proximal landing zone for stent was chosen. The ideal landing zone was between RCCA and RSA. The main body of the single-branched stent, Castor (MicroPort, Shanghai) was placed behind the opening of the RCCA, and the branch stent was accurate position at the RSA. The detailed manipulation technique was reported as previous article [6, 7]. To avoid the risk of endoleak from the origin of LSCA, Tornado ® Embolization Coil (COOK, USA) was employed from left brachial artery (Fig. 3).

#### Strategy 4: Direct repair underwent endoaneurysmorrhaphy

**Fig. 4:**
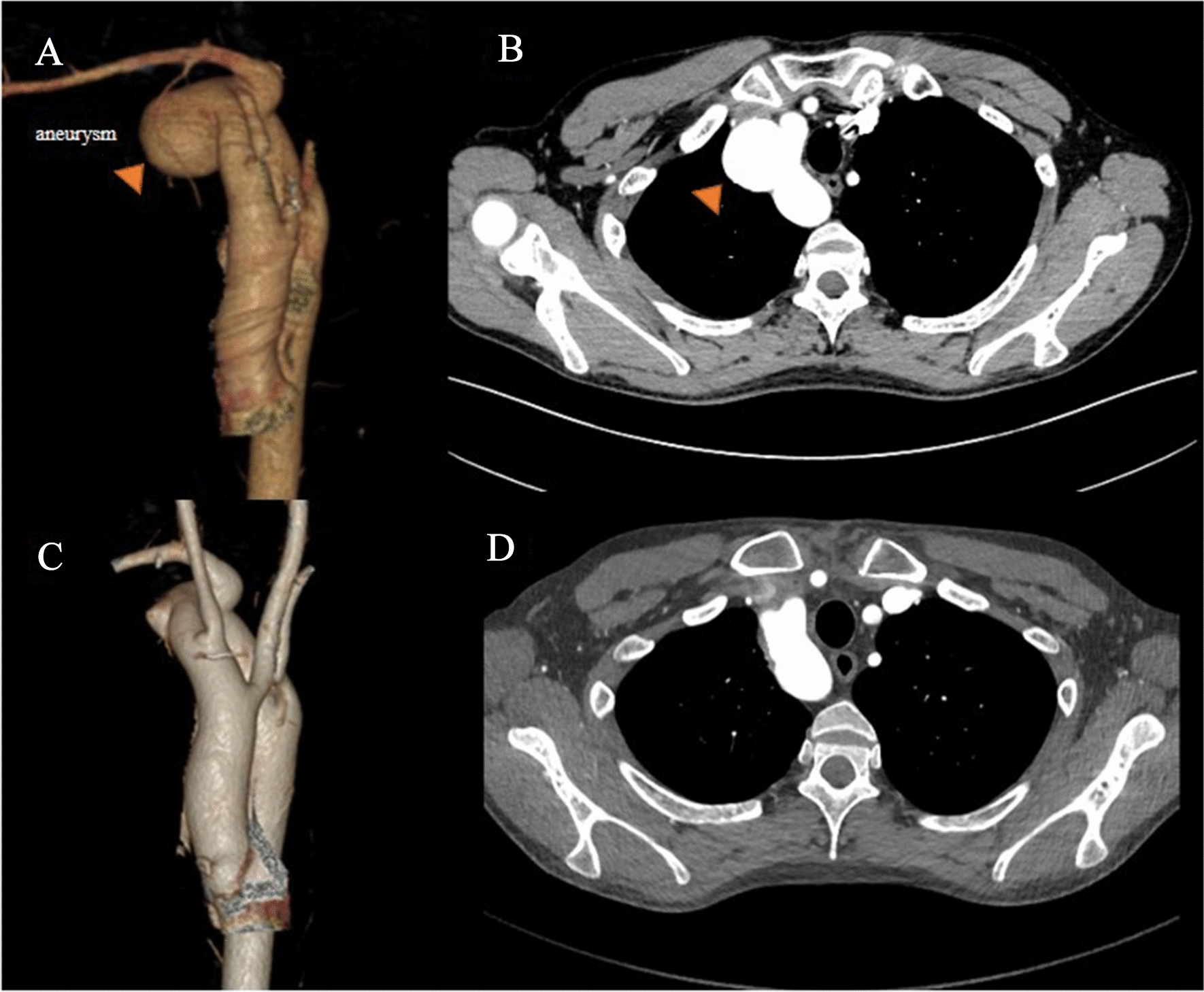
Surgical schema of resection via sternotomy. Preoperative (**A**, **B**) and postoperative (**C**, **D**) three-dimensional reconstruction image of computed tomographic angiogram and CT scan. The dilation aortic aneurysm (orange arrowhead) was resection

Strategy 4 was adopted in a case with small size diverticulum combined with aortic arch aneurysm. After systemic heparinization, CPB was established with ascending aorta and right atrium cannulation. A clamp was placed at the ascending aorta and cardioplegia was infused through the aortic root. Aortic arch was cross-clamp and brain protection was provided by selection cerebral perfusion under the condition of moderate hypothermia (body temperature 26 ℃). Then the aneurysm was opened, and the bottom of aneurysm was closed with artificial patch by placing continuous sutures of 5–0 polypropylene sutures (Fig. 4).

## Results

**Fig. 5:**
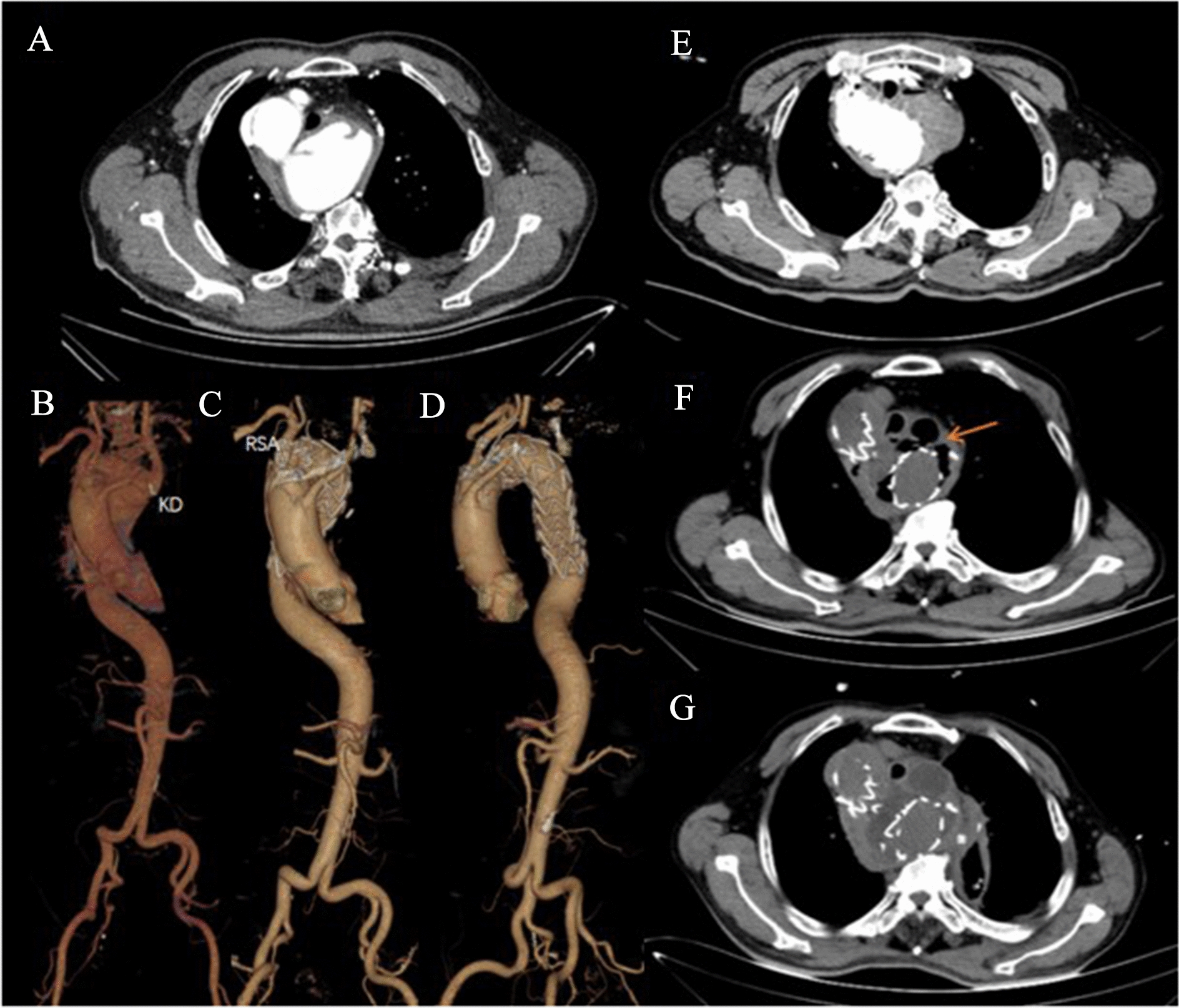
Postoperative complications in patient 3. CT scan and three-dimensional reconstruction image of postoperative computed tomographic angiogram (**A**–**D**) and Patient 3 developed postoperative esophageal fistula complications: 1 week after castor stent implantation (**E**). Esophageal and mediastinal fistula (orange arrow) occurred 1 month after operation (**F**). CT scan after gastroesophagostomy (**G**)

The mean age of these 10 patients was 56.5 years (range 29–79 years) and only 1 woman. Comorbidities included hypertension (*n* = 5), coronary artery disease (CAD; *n* = 1), splenectomy (*n* = 1), gastric ulcer (*n* = 1) and acute coronary syndrome (ACS; *n* = 1). All patients had abnormal aortic arch branching, more rarely, in patient 1; the LSA and LCCA both originated from the Kommerell’s diverticulum. We observed the mean KD size was 41.4 [24.2–56.8] mm. Patients 4, 6 and 8 were operated on under cardiopulmonary bypass (CPB). The mean CPB time was 185.3 [151–210] min. The mean cross-clamping time (CCT) and hypothermia cardiac arrest (HCA) time were 47.3 [37–53] and 37.3 [26–47] min. In addition, patient 8 was the only patient whose diverticulum was not repaired during surgery, and was revealing stable dimensions of the KD with regular CTA follow-up. And the median hospital stay was 22 [15–43] days. There were no in-hospital deaths, but it is regrettable that patient 3 died due to esophageal perforation and mediastinal infection on 2 month after surgery because of esophageal fistula caused by compression of arched hematoma (Fig. 5). And during the follow-up period, patient 1 had a noncardiac sudden death 3 years after surgery. Patients 4 and 6 were readmitted to the hospital underwent two-stage hybrid repair: (1) bilateral carotid and subclavian artery bypass via median sternotomy (strategy 1); (2) at about 3 months after first surgery, they underwent TEVAR via femoral approach as a second-stage procedure. Also, patient 9 developed postoperative hypertension. The mean follow-up period was 21.4 month [range 1–44]. And none of the patients had endoleak during the follow-up period. The detailed information of patients is found in Tables 1 and 2.

**Table 1:** Clinical characteristics of 10 patients who has a congenital anomaly of the aortic arch combined with KD

Patient number	Gender	Age (years)	Smoke	symptoms	Hypertension	Aortic disease	Outcomes	Follow-up (month)
1	M	58	No	Chest pain	Yes	TAA + TBAD	Death	44
2	M	51	Yes	Asymptomatic	No	TAA	Alive	22
3	M	65	No	Back and abdomen pain	Yes	TAA + TBAD	Death (esophageal fistula)	1
4	M	53	Yes	Asymptomatic	Yes	TAA + TBAD	2-stage (rehospitalization)	32
5	F	55	No	Asymptomatic	No	TAA	Alive	25
6	M	58	Yes	Chest and back pain	Yes	TAA + TBAD	2-stage (rehospitalization)	16
7	M	79	No	Chest pain	No	B-IMH	Alive	16
8	M	29	No	Asymptomatic	No	TAA	Alive	24
9	M	64	No	Chest discomfort	Yes	TAA	Alive	25
10	M	50	Yes	Chest pain	No	TAA + TBAD	Alive	9

**Table 2:** Operative details and outcomes of these patients who repair of ALSCA with Kommerell diverticulum by different surgical techniques

Patient number	KD size	Brand of main body stents	Anatomy	Intervention strategy	Left CS bypass	CPB	CCT	HCA
1	47.9 mm	Lifetech Ankura 32 mm × 200 mm	RCCA–RSA–brachiocephalic trunk	2	Yes			
2	56.8 mm	Castor C322612 Lifetech Ankura 32 mm × 160 mm	LCCA–RCCA–RSA–ALSA	3	Yes			
3	33.7 mm	Castor C383210-2002510	LCCA–RCCA–RSA–ALSA	3	No			
4	48.5 mm	CRONUS ® 30 mm × 100 mm	LCCA–RCCA–RSA–ALSA	1	Yes	210	53	39
5	47.7 mm	Castor C322608-2002515	LCCA–RCCA–RSA–ALSA	3	Yes			
6	42.6 mm	CRONUS ® 30 mm × 100 mm	LCCA–RCCA–RSA–ALSA	1	Yes	195	52	47
7	27.6 mm	Castor C383212 Lifetech Ankura 28 mm × 160 mm	LCCA–RCCA–RSA–ALSA	3	Yes			
8	24.2 mm	–	LCCA–RCCA–RSA–ALSA	4	No	151	37	26
9	42.5 mm	Castor C342810-2002530 CRONUS ® 34 mm × 160 mm	LCCA–RCCA–RSA–ALSA	3	Yes			
10	42.5 mm	Castor C363010	LCCA–RCCA–RSA–ALSA	3	Yes			

## Discussion

As we know, a diverticulum originates from the subclavian artery and causes dilatation of the initial portion of the descending aorta, called the Kommerell’s diverticulum (KD) [2]. An aberrant left subclavian artery with associated KD is an uncommon congenital aortic arch anomaly. Patients with a KD have an increased risk of aortic dissection and rupture. In our study, it was found that the aortic lesions associated with KD occurred under different conditions, such as dissection or aneurysm. Kommerell’s aneurysm is often located at the origin of the aberrant LSCA, and this can also be the site where dissection’s proximal tear originates. Aortic dissections with KD are usually symptomatic, with aneurysmal dilation and compression of adjacent structures. In addition, the risk of dissections and ruptures tends to increase as the KD grows in size [4, 8]. However, the coexistence of TBAD with ALSA and KD is extremely rare, with only a few cases reported. In this research, five patients were identified as having TBAD. With the exception of patient No.4, all of them exhibited varied degrees of chest and back pain but no overt signs of compression.

In view of the diversity as well as the rarity of right aortic arch dissections and aneurysms, no guidelines exist regarding standard treatment. The vast majority of surgeons agree that symptomatic patients should be treated with surgery; however, how to manage asymptomatic patients is still up for debate. The EACTS/ESVS guidelines indicated that aneurysmatic aberrant subclavian arteries ≥ 3 cm in diameter should be considered for repair [9]. Previous studies have shown that patients with a right aortic arch and ALSCA were more often treated with open surgery, which requires either a sternotomy or a right thoracotomy but can result in high mortality. Although there are few literature reports, case reports to date have continued to be emerge with improved surgical methods. Current options include open surgery, TEVAR and hybrid techniques [10–12]. Our treatments in this research mainly addressed various techniques in the current relevant field. One of the four patients who underwent median sternotomy experienced further interventional therapy (patient No.1) and the rest of the six patients selected TEVAR therapy via femoral approach with or without subclavian incision revascularization. The choice of open arch reconstruction or a hybrid approach with open cervical artery revascularization and TEVAR was based on the patient’s preference and the treating surgeon’s judgment of the proximal landing zone’s adequacy. Some authors proposed that KD involving the aortic arch and descending thoracic aorta (DTA) repair could be performed as a two-step procedure, consisting of an arch replacement with frozen elephant trunk graft on CPB and a TEVAR endograft through femoral access [13], and consistent with our approach, patients No. 4 and No. 6 who had a TBAD with Kommerell aneurysm successfully used this stage approach without any complications over the follow-up period. In particular, patient No. 8 who was asymptomatic and had an aneurysm localized to the arch without involving the branch vessels and the diameter of the left subclavian artery diverticula was only 24.2 mm, we decided to perform a watchful waiting strategy for Kommerell’s diverticulum, and hence only an endoaneurysmorrhaphy was pursued. Compared with traditional open surgery, TEVAR [14] is now a common treatment method with the advantages of being less invasive, causing fewer postoperative complications and reducing mortality. According to the Edward [15] classification, the right aortic arch is divided into three types: type I, with mirror-image branching of the three major arteries; type II, with an aberrant subclavian artery; and type III, with isolation of the subclavian artery. In our report, most of these patients had type II variations with only 1 patient (patient No. 1) having type I variations. For patient No. 1, on account of his special abnormal anatomy, both the LCCA and LSCA arise from an enlarged descending arch diverticulum, and to create a prolonged proximal landing zone, we found the LSCA and LCCA had to be covered. Therefore, for this complex variation, in the absence of early surgical experience in our department, we successfully performed branch vessel bypass via median thoracotomy, and then released the stent from the femoral approach to cover the arch dissection. For type B dissection involving the aortic arch, Rihito [16] has reported successful use of a single-stage hybrid operation, which consisted of elephant trunk insertion without arch replacement with TEVAR to prevent a high risk of rupture. Fortunately, in this patient with type B dissection who had a rare right-sided arch with mirror branching, his postoperative course was uneventful and without any complications during the 44-month follow-up period until his natural death.

As we know that the proximal landing zone (PLZ) needs to remain at least 1.5 cm [7], if the landing zone is insufficient, covering the LSCA is inevitable to avoid stent-graft migration. In addition, studies have used longitudinal data to determine whether patients with LSA coverage experienced a significantly higher incidence of stroke than patients who received LSA revascularization during TEVAR [17]. To maintain blood flow through the LSCA, some authors [18, 19] reported a novel approach called the chimney stent for type II variants, which required LSCA revascularization in the previous literature. However, the main concern surrounding this technique is type I endoleak, especially, it might cause a type III endoleak during in situ fenestration. Recent research [14, 20] has found that the castor device, which has a unibody design, allows for accurate release to better assist in LSCA reconstruction and reduced the incidence of endoleaks. Using this approach, researchers have been able to repair various aortic diseases. Fang and his co-workers [7] confirmed the efficiency and safety of castor stents in 134 patients with TBAD or IMH after 30-day early-term follow-up. Subsequently, Ben [21] demonstrated the long-term efficacy of the castor device in aortic ulcers with more than 1-year follow-up. In a multicenter clinical study [6] involving 73 patients, a 5-year survival rate of 93.2% and only 5% endoleaks were found, which also proved the suitability of castor for endovascular treatment. In our cases, the technical success rate was 83.3% (5/6), and there was no in-hospital death, only one died after 30 days of surgery due to esophageal fistula. There is little doubt, however, that there was no endoleak after stent release and the operation was successful according to the postoperative CT scan. This may be due to the formation of the vascular ring in such a right arch patient, the trachea and esophagus might be compressed by the diverticulum and delivering an endovascular stent-graft might still worsen the damage [22]. Although there are few reports on the complications of esophageal fistula after stent implantation, it may be more worthy of our attention to determine whether preventive measures should be taken on the choice of stent size and hematoma absorption.

The limitations of the study are as follows: small sample size and the lack of guidelines in the selection of different surgical techniques. Given the rarity of dissections and aneurysms of the right-side arch with a KD, this is yet inevitable. To sum up, each of the procedures we have mentioned here has its advantages and disadvantages. Combining with approaches that ensure an adequate proximal landing zone, whether open surgery, TEVAR with a femoral approach or a staged hybrid approach could effectively fix aortic dissections, but careful and individualized selection is required.

## Conclusions

Overall, this study strengthens the idea that the right aortic arch with Kommerell’s diverticulum can be treated in different ways, and the Castor stent, which is a new device that has been applied clinically in recent years, has also been proven to be a highly effective and feasible treatment for type B aortic dissection or aneurysm. However, more experience and a longer follow-up are required.

## Data Availability

Data are available on request.

## Abbreviations

KD: Kommerell’s diverticulum
TEVAR: Thoracic endovascular aortic repair
LSCA (LSA): Left subclavian artery
ALSCA: Aberrant left subclavian artery
LCCA: Left common carotid artery
RCCA: Right common carotid artery
RSA: Right subclavian arteries
TAA: Thoracic aortic aneurysm
TBAD: Type B aortic dissection
B-IMH: Type B intramural hematoma
CPB: Cardiopulmonary bypass
CCT: Cross-clamping time
HCA: Hypothermia cardiac arrest
CAD: Coronary artery disease
ACS: Acute coronary syndrome
PLZ: Proximal landing zone
